# The untapped potential of ascites in ovarian cancer research and treatment

**DOI:** 10.1038/s41416-020-0875-x

**Published:** 2020-05-08

**Authors:** Caroline Elizabeth Ford, Bonnita Werner, Neville Frederick Hacker, Kristina Warton

**Affiliations:** 1 Gynaecological Cancer Research Group, Lowy Cancer Research Centre and School of Women’s and Children’s Health, Faculty of Medicine, University of New South Wales, Sydney, NSW Australia; 2 Faculty of Medicine, University of New South Wales, Sydney, NSW Australia

**Keywords:** Ovarian cancer, Ovarian cancer

## Abstract

The build-up of fluid in the peritoneal cavity—ascites—is a hallmark of ovarian cancer, the most lethal of all gynaecological malignancies. This remarkable fluid, which contains a variety of cellular and acellular components, is known to contribute to patient morbidity and mortality by facilitating metastasis and contributing to chemoresistance, but remains largely under-researched. In this review, we will critically analyse the evidence associating ascites with metastasis and chemoresistance in ovarian cancer and provide an update on research in the field. We will argue the case for ascites as a unique and accessible substrate for tracking tumour progression and for translational research that will enhance our understanding of this cancer and lead to improvements in patient outcomes.

## Background

Ascites is the pathological accumulation of fluid in the peritoneal cavity and occurs frequently in hepatic cirrhosis and a number of malignancies.^1^ As a comorbidity, ascites can have deleterious effects on a patient’s quality of life, as it is commonly accompanied by dyspnoea, abdominal tenderness and pain, nausea, anorexia, fatigue and impaired movement.^2,3^ It is most frequently associated with ovarian, pancreatic, colorectal, liver and endometrial cancers, and is consequently known as malignant ascites.^4^ This review will focus on the role of ascites in ovarian cancer and its potential as a unique substrate to track tumour evolution and progression.

Ovarian cancer is the most lethal gynaecological malignancy, with more than 125,000 women dying from this disease every year worldwide. This figure has been predicted to rise by 67% to >250,000 women by the year 2035.^5^ The high mortality rate associated with ovarian cancer is attributed to its advanced stage at the time of diagnosis and the lack of available targeted therapies. Ovarian cancer is not a single disease: there are multiple histological and molecular subtypes that involve different cells of origin and varying patterns of progression and response to therapy.^6,7^ The most common and aggressive subtype of ovarian cancer is high-grade serous ovarian cancer (HGSOC). Ascites is present in more than one third of ovarian cancer patients at initial diagnosis and in almost all cases of relapse.^3,8,9^ The greater the volume and frequency with which ascites accumulates in individual patients, the worse the prognosis.^3^ Although this poor prognosis is thought to be due to its tendency to present with HGSOC and in advanced stage disease (both independent predictors of poor prognosis), notably, ascites is known to contribute to chemoresistance, metastasis and decreased resectability.^8–11^

Patients with advanced ovarian cancer typically undergo debulking surgery to remove the primary tumour and all metastatic foci. If ascites is extensive at presentation, neoadjuvant chemotherapy is usually used to reduce levels and to decrease postoperative morbidity at the time of an interval debulking.^12^ Secondary cytoreduction might be indicated in selected patients^13^ but is uncommon; therefore, the opportunity to study tumour evolution by sampling cells from the solid tumour is limited. However, most patients who present with advanced disease will eventually develop resistance to chemotherapy, and most will develop ascites, which will need repeated paracenteses for palliation.^14^ The presence of ascites therefore provides a unique opportunity to repeatedly sample tumour cells from ovarian cancer patients. As ascites is also considered to have a key role in the metastatic process in ovarian cancer, by investigating its components we might learn more about the process of tumour cell dissemination, opening up opportunities for intervention and improvement in patient outcomes, as we will outline in this review.

## Ascites: what is it and how does it arise?

**Fig. 1:**
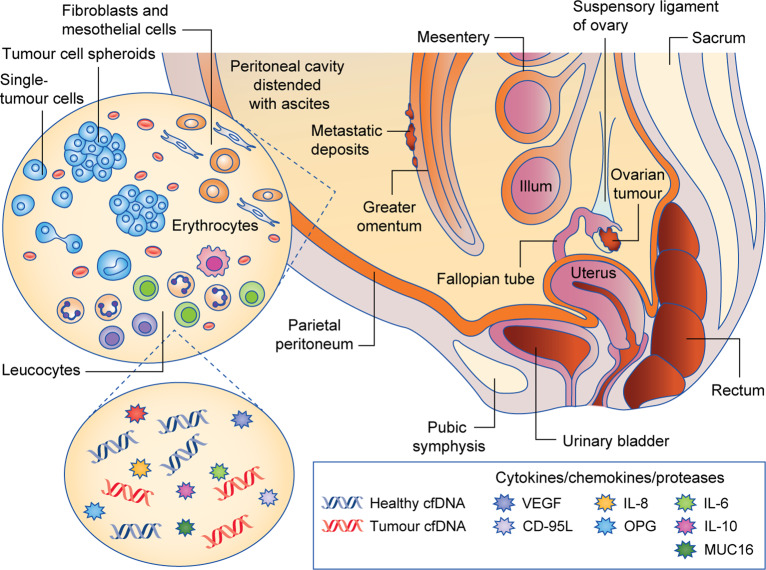
Abdominal accumulation of ascites fluid in ovarian cancer patients. The fluid contains tumour cells, non-tumour cells, circulating free DNA (cfDNA) and signalling molecules. CD-95L CD-95 ligand, OPG osteoprotegerin.

Ascitic fluid contains a range of tumour and non-tumour cells, including fibroblasts, adipocytes, mesothelial, endothelial and inflammatory cells,^15^ as well as cell-free DNA and numerous signalling molecules that mediate cell behaviour (Fig. 1).

**Fig. 2:**
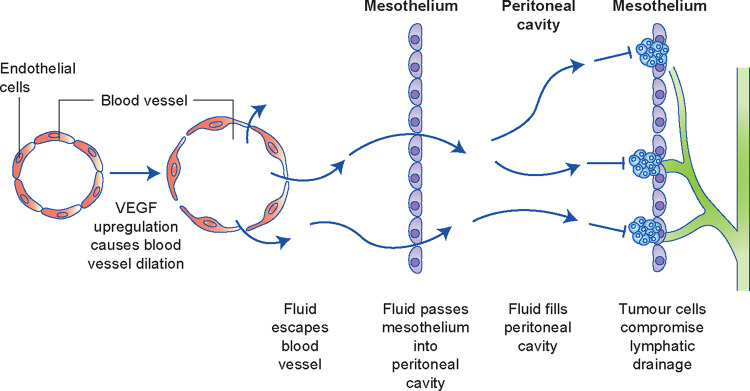
Mechanism of ascites build-up in ovarian cancer. Increased vascular permeability and impaired drainage drive the accumulation of ascites fluid.

The pathogenesis of ascites in ovarian cancer is complex and multifactorial, but it is generally agreed that ascitic fluid build-up occurs if fluid production is heightened, which is facilitated by increased capillary permeability (largely driven by the upregulation of vascular endothelial growth factor (VEGF)), or if the lymphatic drainage capacity of the abdomen is compromised owing to the obstruction of lymphatic stomata in the peritoneum by tumour cells^1,2,16,17^(Fig. 2).

## Ascites and ovarian cancer prognosis

The most important factor that influences patient prognosis in ovarian cancer is the resectability of the primary tumour and metastatic deposits—survival is optimal in cases where the cancer is optimally debulked.^18–20^ Ascites has been shown to increase the chance of suboptimal cytoreduction and is significantly associated with shorter progression-free survival (PFS) and overall survival (OS).^13,21^ Conversely, a number of studies looking at long-term survivors of ovarian cancer have associated the absence of ascites with long-term survivor status.^22–24^ The presence of ascites is recommended to be included in a prognostic nomogram for the prediction of OS in patients with platinum-resistant ovarian cancer.^25^

### Ascites association with subtype, stage and grade of ovarian cancer

Most ascites research focuses on HGSOC, probably due to its prevalence and aggression, but ascites can be present in all subtypes of ovarian cancer, including low-grade serous ovarian cancer (LGSOC) associated with shorter PFS,^26^ clear cell ovarian cancer,^9,27^ mucinous ovarian cancer^9,27^ and endometrioid ovarian cancer.^9^ Unfortunately, in most published studies that include multiple subtypes of ovarian cancer and an association between ascites and OS and/or PFS, the analysis is not stratified according to subgroup, probably due to small cohort sizes, and it is therefore currently difficult to determine the importance of ascites in the progression of some of the rarer subtypes of ovarian cancer.

Although ascites volume was not specifically included as a clinical parameter in the key genome-wide gene expression studies that identified the molecular subgroups of HGSOC,^6,7^ a smaller study of 149 cases of HGSOC did conclude that a subgroup of patients with low-volume ascites defined by the upregulation of immune-related genes and tumour-infiltrating cells might exist.^28^ This group corresponds to the immunoreactive/C2 subgroup defined by the The Cancer Genome Atlas (TCGA).^6^ A lower ascites volume in tumours with an immunoreactive phenotype was also observed in a series of 65 HGSOC cancers.^29^ This suggests that a strong immune response may be important in controlling ovarian-cancer-associated ascites.

The presence of ascites is significantly associated with the extent of disease, with ascites present in >90% of patients with stage III and IV ovarian cancer.^30^ Although the association of ascites with cancer stage is clear, the association with grade is less well defined, as it has not been studied in less frequent subtypes of ovarian cancer. A small number of studies have compared LGSOC and HGSOC and shown a significant difference in the presence of ascites between the two grades, with a higher prevalence of ascites associated with high-grade disease.^9,31,32^

### Ascites volume

A number of studies have also investigated the association of the volume of ascites at surgery with surgical outcomes and patterns of recurrence. Each study has set their own benchmark for high-volume and low-volume ascites. An early study with a relatively high cut-off of 1.8 l reported that patients with higher volumes of ascites had a significantly shorter OS.^30^ This was confirmed in a large study of 685 patients, which showed that patients with an ascites volume >2 l had a significantly shorter PFS and OS.^27^ A 2019 analysis of 210 HGSOC patients found that those with low-volume ascites (defined as <200 ml) had lower levels of CA125, better surgical outcomes and a longer time to recurrence than patients with high-volume ascites (defined as >1 l).^33^ The studies published to date have mostly examined the volume drained at a single time point, as this parameter is easily retrieved from clinical records. However, combining the volume of ascites drained with the frequency of paracentesis over the course of cancer progression would more accurately reflect the rate of abdominal fluid increase, and might add to the accuracy of ascites volume as a prognostic marker.

### Cells and molecules

Individual components of ascites have also been implicated in prognosis. These components include the number and type of cells, as well as signalling molecules, that are present. As discussed later in this review, aggregates of tumour cells that form clusters/spheroids within the fluid might contribute to chemotherapy failure and poor prognosis.^34–38^ A 2012 study recognised the presence of an increased proportion of spheroid cells in the ascites of chemoresistant patients when compared with chemo-naive patients (95% versus 25%).^38^ The presence and populations of immune cells also influences prognosis. A low CD4/CD8 ratio^39^ and high numbers of CD8^+^ effector memory T cells mediated by CXC motif chemokine ligand 9 (CXCL9)^40^ have been reported to be associated with longer PFS, underlining the link between the cell interactions that take place in ascites and progression of the disease.

Tumorigenic cytokines, including pro-inflammatory interleukin (IL)-6, IL-8 and tumour necrosis factor (TNF), as well as VEGF, have been detected in ascites, with increased levels of these cytokines being linked to shorter PFS,^41,42^ although the effect of IL-6 has not been observed consistently across studies.^43^ Conversely, high levels of the anti-inflammatory cytokine IL-10 have been associated with longer survival.^44^

It would be useful to investigate how ascites-derived signalling molecules and cell profiles change over the course of chemotherapy and in patients in remission versus relapsed patients. This information might identify prognostic markers that indicate the likelihood of relapse. Data from such an investigation might give valuable insight into how specific phenotypic distributions of suspended malignant cells influence treatment outcomes and might, in the future, become an indication for different treatment strategies.

## Ascites and metastasis

While metastasis from ovarian cancer can occur via a haematogenous route^45^ or lymphatic routes, most ovarian cancers primarily spread across the peritoneal cavity. This transcoelomic spread facilitates a more efficient process of metastasis, as malignant cells follow the dynamics of the peritoneal fluid to the squamous epithelium that lines the cavity—the mesothelial lining—where they will seed.^16^ This ‘passive’ metastasis results in the distribution of cellular deposits preferentially in areas where fluid accumulates within the peritoneum when in the supine position (the Pouch of Douglas and right subphrenic region), as well as in areas with constant and extensive exposure to peritoneal fluid (the omentum).^46,47^ This facilitated metastasis results in the common occurrence of peritoneal carcinomatosis, a more diffuse and widespread form of metastasis that negatively influences surgical resectability.^48^

Malignant ascitic fluid is rich in tumour-promoting cytokines, chemokines, growth factors and proteinases and, as such, is considered a unique form of the tumour microenvironment, a feature recognised for its importance in metastasis (as well as in chemoresistance, discussed below).^49,50^ Cell-free supernatant extracted from the malignant ascites of ovarian cancer patients has been shown to promote the metastatic process by reducing the strength of tight-junctions (through downregulation of the expression of E-cadherin, connexin 43, occludin, and desmoglein) between mesothelial cells, thereby assisting transmesothelial migration.^11^ There is also broader evidence that ascites is involved in promoting epithelial-to-mesenchymal transition (EMT), by shifting cancer cells towards a stem-cell-like phenotype.^38,51,52^ Cancer stem cells have the capacity to self-renew and differentiate, reducing their vulnerability to chemotherapy, especially in spheroids.^53^ EMT has been consistently implicated as a major contributor to ovarian cancer invasion, metastasis and chemoresistance.^54,55^

## Ascites and chemoresistance

In use for over 30 years, platinum-based drugs remain the most common chemotherapy treatment option following cytoreductive surgery for patients with advanced ovarian cancer.^56^ Patients with ovarian cancer generally respond well to chemotherapy but the tumours often recur, which is a major ongoing clinical challenge. Over 80% of patients with ovarian cancer have recurrent disease after chemotherapy and lack other treatment options.^57^ Recurrence and/or chemoresistance is generally indicated by clinical symptoms, including the development of ascites, an increase in CA125 levels, or radiological evidence of the presence of disease by CT or PET-CT scans.

The development of ascites while receiving chemotherapy, or shortly after completing a treatment cycle, is considered to be a poor prognostic marker and evidence of the likely development of chemoresistance. However, it is unclear if ascites is merely a symptom of failing chemotherapy, or if components of the ascites itself are responsible for the development of chemoresistance.

### Cell spheroids

**Fig. 3:**
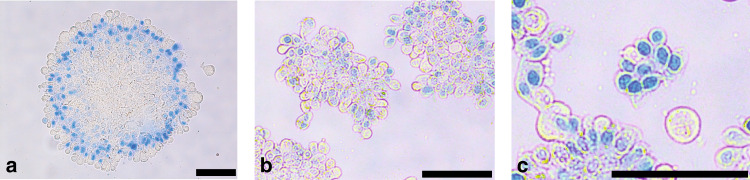
Three different sized spheroids isolated from a patient with low-grade serous ovarian cancer (LGSOC). **a**) large spheroid **b**) medium spheroid **c**) small spheroid. Spheroids stained with Trypan Blue. Black bar = 100 µM. Unpublished data (B.W.).

One potential contributing factor to the association between ascites and chemoresistance is the presence of highly tumorigenic cell spheroids.^30,33^ Cell spheroids are aggregates of cells (both cancer and non-cancer cells) that exist in, and can be isolated from, ovarian-cancer-associated ascites. Spheroids can range in size and structure (Fig. 3). Model systems of spheroids have been shown to limit the efficacy of classic cytotoxic drugs and restrict the access of chemotherapeutics.^36,49^ One study demonstrated up to fourfold higher resistance to cisplatin in a spheroid population compared with a single-cell population of ovarian cancer cell lines.^35^ Quiescence within spheroids, due to hypoxia in the deeper core cells, has also been postulated to be involved in chemoresistance.^58,59^ However, other findings suggest that the core of spheroids might in fact be dominated by mesothelial cells, rather than tumour cells,^60^ so further research is required to clarify how targeting spheroids may or may not be a useful therapeutic strategy. Differences in DNA stability, gene transcription and epigenetic patterns between spheroid and monolayer ovarian cancer cells have also been identified, which might indicate differences in drug sensitivity and metastatic potential; however, the actual significance of these differences is yet to be determined.^61^

### Signalling molecules

There is growing evidence that non-tumour components of ascites are significant in driving chemoresistance.^62^ For example, cholesterol in ascites upregulates the expression of the multidrug resistance protein 1 (MDR1) efflux pump in ovarian cancer cell lines,^63^ whereas cholesterol depletion inhibits cisplatin resistance.^63^ However, cholesterol depletion did not affect resistance to paclitaxel in most cell lines studied. Furthermore, no assays were performed combining both therapies. As such, the significance of cholesterol-induced cisplatin resistance in a situation consistent with real-world application is yet to be shown.^63^

In addition, a number of key pathways have been implicated in in vitro and in vivo studies of ovarian cancer ascites, including focal adhesion kinase (FAK), AKT, extracellular signal-regulated kinase 1/2 (ERK1/2) and IL-6 production.^64–66^

## Targeting ascites production as a treatment option for ovarian cancer

Ascitic fluid has been described as a prerequisite for the characteristic transcoelomic metastasis of ovarian cancer by facilitating the dissemination of tumour cell spheroids and acting as a growth-promoting medium.^67,68^ It has also been implicated as a promoter of lymphatic metastasis and subsequently as an avenue for haematogenous spread.^69,70^ As such, it is worth considering whether preventing ascites might be useful in combating the disease.

At present, ascites and its symptoms are managed by performing paracentesis when required. No other strategies are currently implemented into standard practice to treat or prevent ascites production secondary to ovarian cancer, but a potential way to inhibit ascites could be via anti-angiogenic therapy. VEGF has been consistently implicated in ascites production by reducing the strength of tight-junctions between peritoneal endothelial cells, thereby enhancing the permeability of the endothelium,^71,72^ and inhibiting VEGF has been demonstrated to control ascites.^73^ The anti-angiogenic therapy bevacizumab, an established monoclonal antibody targeting VEGF, has therefore been investigated in ovarian cancer clinical trials as both frontline therapy and at disease relapse.^74^ In Phase 3 clinical trials, bevacizumab improved PFS when used in conjunction with the conventional treatment regimen.^75–78^ However, clinical trials of bevacizumab to date have focussed on its effect in inhibiting angiogenesis and starving the tumour, without regard for any incidental benefit of reducing ascites. A landmark trial of bevacizumab, AURELIA, reported the control of ascites in a subgroup of participants with platinum-resistant recurrent ovarian cancer with ascites at baseline.^77^ The subgroup was reported to have an improved PFS, but not more so than in patients without ascites. However, as the disease stages of patients with ascites were not specified, it is unclear whether patients with earlier intervention and less chronic ascites might have benefitted more than those with already extensive disease. As it is, the improvement in PFS is noteworthy, given the subgroup’s poor prognosis, and demands further investigation into the most optimal time to administer bevacizumab to patients with, or at risk of developing, ascites.

Limited approaches have been taken towards targeting angiogenesis with the primary intention of inhibiting ascites production and, although these approaches were proven to be effective in reducing ascites, the experimental designs were directed towards the palliative management of advanced staged disease with symptomatic, chronic ascites, rather than early intervention.^73,79^ A 2019 review describing the indication criteria for the recommendation of bevacizumab did not mention ascites, highlighting that this therapy is still under-researched in this area.^80^ However, despite the targeting of ascites production not yet being investigated as a primary approach to delaying disease progression, the existence of an effective and Food and Drug Administration (FDA)-approved ascites-controlling first-line therapy (bevacizumab) presents an opportunity to explore the impact of addressing this disease pathway.

## Ascites as a liquid biopsy substrate

The molecular analysis of patient clinical samples is often hampered by the small amounts of material available, limiting the accuracy and number of assays that can be undertaken. Ascites is an exception in this regard, in that it is not unusual for large volumes to be removed from patients, often repeatedly, thus essentially representing a consecutive sampling of the milieu in which ovarian cancer spreads. This makes ascites an ideal medium for analysing both the response to therapy and the development of chemoresistance.

### Monitoring therapeutic response

Limited research has been directed towards utilising ascites to monitor the response to therapy, but emerging evidence supports the future possibility of this approach. Tumour-associated autoantibody signatures that are specific for ovarian cancer have been identified in ascites and found to correlate with response to first-line therapy.^81^ A profile of autoantibodies against the tumour-associated antigens BCL6 corepressor (BCOR), mitochondrial ribosomal protein L46 (MRPL46) and cAMP-responsive element-binding protein 3 (CREB3) was shown to significantly decrease in ascites from platinum-resistant patients versus platinum-sensitive patients.^81^ Unfortunately, the signature was not able to significantly differentiate the extent of resistance; however, the potential use of this approach has been illustrated.

Another opportunity offered by the presence of ascites is the ability to test and predict drug response based on the interaction of a selected therapy with ascites tumour cells within their respective tumour microenvironment. Cell spheroids isolated from patients ascites samples have previously been demonstrated to reflect the response to therapy, identifying them as a viable candidate for drug-screening methods.^82^ Soluble components of the fluid have also been demonstrated as predictors of response, with high levels of insulin-like growth factor (IGF)-I inversely correlating with objective clinical response to neoadjuvant chemotherapy.^83^ A new method to grow ovarian cancer organoids derived from single cells isolated from ovarian-cancer-associated ascites has been described.^84^ A comparison of expression profiles from five ovarian cancer patients using single-cell RNA-sequencing on clinical samples across primary tumours and metastatic sites revealed that the ascites-derived organoids retained the molecular diversity of the patient and therefore could act as ‘patient-matched avatars’ for a precision oncology approach to treatment. Other groups have reported growing tumour cells from ascites in vitro for drug sensitivity testing,^85^ and predicting clinical resistance via evaluation of biomarkers in the ascites.^86^

### The development of chemoresistance

Tumour cells that are present in ascites can provide a substrate for analysing mutations that are involved in the development of chemoresistance. Patch et al.^87^ used ascites to identify reversions in BRCA1 and BRCA2 mutations and decreased methylation that underlie loss of sensitivity to poly-ADP ribose polymerase inhibitors, as well as promoter gene fusions that drive overexpression of the MDR1 drug efflux pump. In addition, gene expression profiles that are linked with chemoresistance have been described in ascites tumour cells: the expression of the stem-cell markers Oct4, EpCam and CD44 has been shown to be upregulated in ascites tumour cells from chemoresistant samples,^38^ and might link the stem-cell properties of resistant samples with increased efficiency in drug efflux.^88^

Ascites provides a highly suitable medium for tracking cancer clones that are in the process of developing drug resistance, as it captures cells that are more broadly representative of the assortment of tumour deposits than biopsy samples from a single site or even multiple solid tissue sites. Sampling ascites might allow for a more comprehensive assessment of a patient’s mutation profile, which will continue to gain importance as targeted drugs and personalised medicine advance.

Malignant ascites has been found to contain tumour-derived circulating free DNA (cfDNA) in addition to tumour cells.^89^ Like tumour cells within ascites, cfDNA presents an opportunity for non-invasive tumour genome analysis without the bias of sampling a limited number of biopsy sites.^15^ If tumour cell numbers in ascites are low, cell-free tumour DNA (ctDNA) could offer additional material for mutation analysis. Although there has been an explosion of interest and research into the opportunity that circulating tumour cells and ctDNA provide as liquid biopsy samples from blood,^90,91^ as yet relatively little attention has been given to other fluids, including pleural effusions and ascites. Currently, patients are primarily monitored for the recurrence of ovarian cancer after treatment by measuring the level of CA125 in their blood. However, initial data suggest that ctDNA might be more sensitive than CA125 in detecting recurrence,^92^ although the ctDNA fraction in patients with low (ascitic fluid) volume disease can be insufficient for extensive profiling of mutations, and the blood volumes that can be sampled are limited. The proportion of ctDNA in ascites fluid has not been described; however, as the fluid is present in the immediate vicinity of the tumour and contains suspended cancer cells, the tumour fraction is likely to be higher than that in blood plasma and, furthermore, the volumes of ascites available for analysis are vastly larger than is feasible from blood sampling. This suggests that when patients present with ascites, ctDNA in the fluid could be used to track changes in the mutation profile that accompany resistance to therapy and disease recurrence.

It remains important, however, to consider how representative tumour cells in the ascites are of the primary tumour, and whether ascites can truly be used to track tumour evolution. A number of studies have attempted to address this question. Kim et al.^93^ used whole-exome sequencing to analyse ten spheroids derived from ascites and eight primary tumour samples from one HGSOC patient to show that the ascites-derived tumour cells were an independent lineage to the primary tumour cells, and suggested this was an early event in tumour evolution and metastasis.

## Identifying new therapeutic strategies by studying ascites

Ascites provides an opportunity to develop the treatment options available to ovarian cancer patients not only through its potential function as a liquid biopsy medium, but also as a substrate in which to identify new drug targets.

One such approach is to target the highly tumorigenic cell spheroids that are nurtured in ascitic fluid. As spheroid tumorigenicity is largely driven by EMT, much focus has been placed on the pathways responsible for driving this phenotypic change.^94^ Various efforts have been made to identify known drivers of EMT in ascites-derived cells that are potentially targetable in order to develop treatments that are complimentary to the current management strategy.^95^ Some examples of this approach are described below.

The efficacy of inhibiting signal transducer and activator of transcription 3 (STAT3), a therapeutic target in a variety of cancers, is currently being assessed.^96^ STAT3 has been reported to be constitutively active in ascites-derived ovarian cancer cells and is implicated in the heightened malignancy of spheroids by instigating EMT via activation of the Wnt signalling pathway.^97,98^ Transplantation of ascites-derived ovarian cancer cells that express high levels of STAT3 into the ovarian bursa of mice induced the formation of a large primary tumour and widespread peritoneal metastases.^80^ By contrast, STAT3 inhibitors reduced chemoresistance and spheroid tumorigenicity^97,99^ in murine models. Other groups have investigated inhibiting the phosphatidylinositol 3-kinase (PI3K)/Akt/mammalian target of rapamycin (mTOR)-mediated EMT pathway by silencing leptin, which is found in abundance in ascites; again, promising results have emerged from murine models.^100^ Transforming growth factor (TGF)-β is another marker of EMT present in ascites that is attracting interest in this area, with various investigations implicating it as a major driver of metastasis with demonstrated potential as a therapeutic target.^94,101^ With a broader understanding of the mechanisms involved in the development and seeding of ovarian cancer spheroids, more potential targets are likely to be identified in the future.

## Challenges and future opportunities for ascites research

Ascites is a uniquely valuable substrate for research for multiple reasons, including its availability—both in volume and frequency—and its simultaneous reflection of both primary and secondary tumours and their microenvironments. As it remains considerably under-researched, the opportunities for growth in understanding this remarkable fluid and how we can best take advantage of it are plentiful.

The potential comprehensive insight this fluid gives into the primary tumour and its evolution towards metastasis provides the outstanding possibility of furthering our understanding of the key mechanics involved in this process and identifying potentially targetable markers. A better insight into the importance of soluble and cellular components of the fluid in driving metastatic change or harbouring tumour growth will be key. As summarised in this review, there is significant evidence that ascites contributes to ovarian cancer metastasis and chemoresistance. However, it is important to note that there is also some evidence of potential tumour-suppressing qualities of ascites in ovarian cancer. An in vitro study identified fibrin/fibrinogen degradation products in ascitic fluid, which showed anti-angiogenic properties.^102^ This study highlights the need to further clarify the role of individual components of this substrate-rich fluid.

Ascites is already showing promise both as a predictor of drug response and as a substrate for monitoring drug efficacy, but much more investigation into its potential is warranted.^81,100,101^ While it remains true that if a patient responds to a given treatment the ascites will resolve and be inaccessible, in the recurrent setting, complete responses to therapy are uncommon, and duration of response is often short, so after an initial resolution, the ascites will reappear. It is true also that with regular monitoring of tumour markers such as CA125, rising titres will usually be observed before ascites develops, but not all patients are diligent about follow-up, and not all clinicians recommend such close follow-up.^103,104^ Hence, the presence of ascites often heralds the presence of recurrent disease. The availability of recurrent samples provides the opportunity to track tumour progression, assess drug response and indicate changes to the existing treatment approach before chemoresistance develops, as it so commonly does in this disease.^102^ Furthermore, the opportunity exists to identify prognostic signatures in the fluid, which could direct the management of the disease on a case-by-case basis, especially as targeted therapeutics begin to emerge for ovarian cancer.

A challenge to be overcome when considering further work with this sample type is the variable capturing of ascites in tumour biobanks and clinical databases.^47^ Ascites and its contents are not routinely stored for downstream analysis in biorepositories, and volumes are often inconsistently recorded. As such, the possibility of retrospective or large-scale analysis of ascites samples is, at this stage, limited. However, an increased awareness of the potential of this substrate for research might encourage more comprehensive documentation and cataloguing of this valuable sample, which will serve to enhance research possibilities as they advance. Another challenge when working with ascites is that sample transfer to researchers might be prone to delay, as the samples are retrieved secondary to a patient’s therapeutic paracentesis. As such, it is crucial to consider how the integrity of the samples is affected by time, to ensure that valid research can be performed. A standardised protocol for handling this unique biospecimen is warranted. Meeting these challenges will allow us to harness the full potential of this ascites and might provide the key for improving outcomes for this devastating disease.

## Data Availability

Previously unpublished examples of ascites spheroids are shown in Fig. 3.
